# Functional up-regulation of Na_v_1.8 sodium channel in Aβ afferent fibers subjected to chronic peripheral inflammation

**DOI:** 10.1186/1742-2094-11-45

**Published:** 2014-03-07

**Authors:** Mounir Belkouch, Marc-André Dansereau, Pascal Tétreault, Michael Biet, Nicolas Beaudet, Robert Dumaine, Ahmed Chraibi, Stéphane Mélik-Parsadaniantz, Philippe Sarret

**Affiliations:** 1 Department of Physiology and Biophysics, Faculty of Medicine and Health Sciences, Université de Sherbrooke, 3001, 12th Avenue North, Sherbrooke, Quebec J1H 5N4, Canada; 2 Pain Group, Centre de Recherche de l’Institut du Cerveau et de la Moelle Épinière, Université Pierre et Marie Curie, INSERM-Unité Mixte de Recherche en Santé 975, CNRS-Unité Mixte de Recherche 7225, 75013 Paris, France

**Keywords:** Aβ-fibers, Allodynia, Complete Freund’s adjuvant, Electrophysiology, Sodium channel blocker

## Abstract

**Background:**

Functional alterations in the properties of Aβ afferent fibers may account for the increased pain sensitivity observed under peripheral chronic inflammation. Among the voltage-gated sodium channels involved in the pathophysiology of pain, Na_v_1.8 has been shown to participate in the peripheral sensitization of nociceptors. However, to date, there is no evidence for a role of Na_v_1.8 in controlling Aβ-fiber excitability following persistent inflammation.

**Methods:**

Distribution and expression of Na_v_1.8 in dorsal root ganglia and sciatic nerves were qualitatively or quantitatively assessed by immunohistochemical staining and by real time-polymerase chain reaction at different time points following complete Freund’s adjuvant (CFA) administration. Using a whole-cell patch-clamp configuration, we further determined both total I_Na_ and TTX-R Na_v_1.8 currents in large-soma dorsal root ganglia (DRG) neurons isolated from sham or CFA-treated rats. Finally, we analyzed the effects of ambroxol, a Na_v_1.8-preferring blocker on the electrophysiological properties of Na_v_1.8 currents and on the mechanical sensitivity and inflammation of the hind paw in CFA-treated rats.

**Results:**

Our findings revealed that Na_v_1.8 is up-regulated in NF200-positive large sensory neurons and is subsequently anterogradely transported from the DRG cell bodies along the axons toward the periphery after CFA-induced inflammation. We also demonstrated that both total I_Na_ and Na_v_1.8 peak current densities are enhanced in inflamed large myelinated Aβ-fiber neurons. Persistent inflammation leading to nociception also induced time-dependent changes in Aβ-fiber neuron excitability by shifting the voltage-dependent activation of Na_v_1.8 in the hyperpolarizing direction, thus decreasing the current threshold for triggering action potentials. Finally, we found that ambroxol significantly reduces the potentiation of Na_v_1.8 currents in Aβ-fiber neurons observed following intraplantar CFA injection and concomitantly blocks CFA-induced mechanical allodynia, suggesting that Na_v_1.8 regulation in Aβ-fibers contributes to inflammatory pain.

**Conclusions:**

Collectively, these findings support a key role for Na_v_1.8 in controlling the excitability of Aβ-fibers and its potential contribution to the development of mechanical allodynia under persistent inflammation.

## Background

The processing of sensory information from primary afferent neurons to the spinal dorsal horn may change significantly following tissue inflammation, ultimately leading to the development of chronic pain. Abnormal pain manifestations, such as allodynia, hyperalgesia, and spontaneous pain episodes occurring in these pathological pain states, are believed to result, at least in part, from plasticity phenomena in the spinal sensory system [1,2]. Functional alterations in the properties of Aβ primary afferents may notably account for the increased pain sensitivity observed under peripheral chronic inflammation. Variations in neurotransmitter content and release, changes in membrane receptor function and trafficking, and regulation of ion channel expression and activity may indeed enhance the excitability of Aβ-fibers and thus contribute to the development of mechanical hypersensitivity following peripheral chronic inflammation [1-3].

There is now considerable evidence supporting the idea that hyperexcitability and spontaneous action potential firing mediated by voltage-gated sodium channels in peripheral sensory neurons play an important role in the pathophysiology of chronic pain [4,5]. Among them, the slow-inactivating tetrodotoxin-resistant (TTX-R) sodium channel, Na_v_1.8, has been pointed as a key contributor in the development of painful sensations associated with chronic inflammation in peripheral tissues [4,6]. Accordingly, several inflammatory mediators acting through G protein-coupled receptors, including adenosine, serotonin, prostaglandins, and chemokines, have been shown to sensitize TTX-R sodium channels and therefore to increase sensory neuron excitability [7-10]. Furthermore, functional knockdown of Na_v_1.8 in rodents and spinal or systemic administration of Na_v_1.8 channel blockers attenuate nociceptive behaviors related to persistent inflammation [4,5,11,12]. Although the Na_v_1.8 channel is localized predominantly in small/medium nociceptive C/Aδ-type dorsal root ganglia (DRG) neurons, Na_v_1.8 is also expressed by large myelinated Aβ afferent fibers in both healthy and inflamed animals [13-25]. We therefore hypothesized that the changes in the biophysical and pharmacological properties of Na_v_1.8 might modulate the excitability of large-diameter sensory neurons under chronic peripheral inflammation.

In the present study, we thus investigated both total I_Na_ and TTX-R Na_v_1.8 currents in large-soma DRG neurons isolated from sham or complete Freund’s adjuvant (CFA)-treated rats, a well-established animal model of chronic inflammatory pain. We further determined whether this persistent inflammation led to alterations in the expression and localization pattern of Na_v_1.8 in Aβ afferent fibers and redistribution in peripheral axons. Finally, we also examined the effects of ambroxol [26], a Na_v_1.8-preferring blocker, on the electrophysiological properties of Na_v_1.8 currents and on the development of mechanical allodynia following intraplantar CFA injection.

## Methods

### Animals and chronic inflammation induction

Adult male Sprague–Dawley rats (200 to 225 g, Charles River, St. Constant, Québec, Canada) were housed two per cage in a climate-controlled room on a 12 h light/dark cycle with water and food available ad libitum. They were allowed at least 5 days to habituate to the housing facility prior to manipulation and 1 hour to habituate to the experimentation room before any behavioral study was performed. All experimental procedures were approved by the Animal Care and Use Committee of the Université de Sherbrooke, and were in accordance with the policies and directives of the Canadian Council on Animal Care and guidelines from the International Association for the Study of Pain.

CFA (Calbiochem, La Jolla, CA, USA) was prepared by complementing it with 7 mg/ml of *mycobacterium butyricum* (Difco Laboratories, Detroit, MI, USA) and emulsified 1:1 with saline 0.9%. Under light anesthesia with isoflurane, rats received an intraplantar injection of 100 μl (400 μg) of the freshly emulsified mixture into the left hind paw. Sham animals received an intraplantar injection of 100 μl of saline.

### Drugs

On days 3, 8, and 14 post-CFA administration, every rat was given an intrathecal (i.t.) injection of ambroxol (0.1 mg/kg, Sigma-Aldrich), a Na_v_1.8-preferring sodium channel blocker or vehicle (DMSO 6%), for a total of three injections per rat [27]. Ambroxol was delivered between the lumbar vertebrae L5 and L6 in a final volume of 25 μl to lightly anesthetized animals with a Hamilton syringe fitted to a 27^½^ gauge needle. The cumulative dose of ambroxol (0.3 mg/kg) was chosen based on previous literature reporting affinity, selectivity, and in vivo profiles of this compound [27-29]. Mechanical sensitivity and edema were then determined (see details below) 1 h after the last administration of the drug. I.t. injection of drug solutions and behavioral testing were conducted on the basis of a blind and randomized design, in which one experimenter took the charge of drug preparation, whereas another experimenter who was blind to drug administration, randomly divided rats into two groups and conducted the measurements of mechanical withdrawal threshold and paw volume. Cultured DRG neurons isolated from rats treated for 14 days with CFA were also incubated for 30 min with ambroxol, applied at two different concentrations (20 and 100 μM) before patch clamp recordings.

### Mechanical sensitivity

The onset and progression of mechanical hypersensitivity over a 21-day period in this CFA model has been previously published elsewhere [30]. Mechanical sensitivity testing was done in all rats prior to collection of tissue samples at days 3, 8, and 14 post-CFA using an electronic von Frey device (Ugo Basile Dynamic Plantar Aesthesiometer, Stoelting, IL, USA). Briefly, a dull metal probe (0.5 mm diameter), placed underneath a mesh floor, was applied against the hind paw pad and triggered when the animals were standing firmly. The probe exerted a ramping pressure of 3.33 g/sec. The force required to elicit a withdrawal response was automatically recorded upon the withdrawal of the hind paw and taken as the index of mechanical nociceptive threshold; the cut-off was set to 50 g. Four stimulations were applied alternately on the CFA-injected ipsilateral and contralateral hind paws. Average ipsilateral and contralateral paw withdrawal thresholds were calculated for each animal. Rats were acclimatized to the device for 3 days before testing. On the 14th day following CFA administration, a chronic inflammation-induced hypersensitivity state was observed.

### Edema

The volume of the hind paw was determined with a plethysmometer (Stoelting (Panlab), IL, USA) in rats treated for 14 days with CFA as well as in sham animals. The inflexion point of the ankle joint was used as an anatomical reference. The water displacement following immersion of the animal’s paw in the measuring tube, into a second communicating tube induces a change in the conductance between the two platinum electrodes. The Plethysmometer Control Unit detects the conductance changes and generates an output signal to the digital display indicating the volume displacement (0.01 ml resolution).

### Quantitative Real-Time PCR (qRT-PCR)

For qRT-PCR analysis, lumbar ipsilateral DRG (L4 to L6) were harvested on day 14 post-CFA injection, 1 h after the behavioral measurement, and then quickly snap frozen in dry ice. Total RNA was extracted using RNeasy® Mini Kits (Qiagen GmbH, Hilden, Germany). Both RNA quantity and quality were analyzed with a NanoDrop® 1000 spectrophotometer (Thermo Fisher Scientific, Wilmington, DE, USA). Reverse transcription of the samples was performed with TaqMan® Reverse Transcription Kits (Applied Biosystems, Carlsbad, CA, USA) using 400 ng of total RNA as template. Real time reactions were processed in triplicate for every cDNA sample on a Rotor-Gene 3000 (Corbett Life Science, Kirkland, Québec, Canada) using TaqMan® Gene Expression Master Mix (Applied Biosystems). Na_v_1.8 levels were normalized against the housekeeping gene GAPDH and analyzed by the relative standard curve method. DNA oligonucleotides and probes used in Taqman assay are listed in Additional file 1: Table S1. The probes were conjugated with fluorescent reporter dyes 6-FAM at the 5’ end and the quencher dye Iowa Black FQ at the 3’ end (Integrated DNA Technologies, Inc., Coralville, IA, USA).

### Axonal transport of Na_v_1.8 in the sciatic nerve and quantification

To visualize the intra-axonal transport of Na_v_1.8, a single ligature was placed around the sciatic nerve proximal to the trifurcation on day 12. Briefly, the left sciatic nerve of sham or CFA-treated rats was exposed at the level of the upper thigh, and tightly ligated with a 4.0 silk suture under deep anesthesia. At 48 h post-ligation, on day 14, 3-mm-long sciatic nerve segments proximal to the ligature were then harvested and processed for histology, as described below. For the quantification, sciatic nerve sections of CFA-injected animals (n = 9) or sham animals (n = 3) were successively photographed with the same camera parameters (Axio Vision; Carl Zeiss, Oberkochen, Germany). The accumulation of Na_v_1.8-immunoreactivity was examined in the sciatic nerve in an area of 1 mm proximal to the ligation site. The same threshold in grey levels was applied to all sections and the percentage of labeling density per fixed area of the Na_v_1.8-immunoreactivity was quantified with Image J (version 1.46r, NIH) and reported as arbitrary units.

### Immunolocalization of Na_v_1.8 in dorsal root ganglia and sciatic nerves

At 3, 8, and 14 days after CFA or sham injection, rats were deeply anesthetized with ketamine (87 mg/kg)/xylazine (13 mg/kg) administered intramuscularly and perfused transaortically with a freshly prepared solution of 4% paraformaldehyde (PFA) in 0.1 M phosphate buffer saline (PBS), pH 7.4. Ipsilateral lumbar L4-L6 DRGs were rapidly removed, cryoprotected overnight in 0.1 M PBS containing 30% sucrose at 4°C, and snap frozen in isopentane cooled at −40°C. Tissues were sectioned longitudinally at a thickness of 20 μm on a Leica CM1850 cryostat. The sections were then processed for indirect immunofluorescence labeling, as previously described [31]. Briefly, serial sections were treated for 30 min at room temperature in a blocking solution containing 2% normal goat serum (NGS) and 0.5% Triton X-100 in PBS, and incubated overnight at 4°C with a mixture of primary antibodies in PBS containing 0.05% Triton X-100 and 0.5% NGS. To detect Na_v_1.8, sections were incubated with the rabbit polyclonal anti-Na_v_1.8 antibody (1:200; Alomone Labs, Jerusalem, Israel) in PBS containing 0.05% Triton X-100 and 0.5% NGS. To identify Na_v_1.8-expressing large sensory neurons, DRG sections were processed for double immunofluorescence labeling with the mouse monoclonal anti-neurofilament 200 (1:400; NF200-clone N52; Sigma, Oakville, ON, Canada). After extensive washing with PBS, bound primary antibodies were revealed by simultaneous incubation with goat anti-rabbit Alexa 488- and goat anti-mouse Alexa 594-conjugated secondary antibodies (1:500; both from Molecular Probes, Burlington, ON, Canada) for 60 min at room temperature. After rinsing, sections were mounted in anti-fade mounting medium for fluorescent microscopy. For specificity control, sections were incubated overnight with primary antiserum pre-adsorbed with the Na_v_1.8 corresponding antigen. The absence of cross-reactivity of the secondary antibodies was also verified by omitting one or both primary antibodies during the overnight incubation. The same procedure was performed for preparing the ligated sciatic nerves on day 14.

### Image acquisition and analysis

Labeled sections were examined under fluorescent illumination with a Leica DM-4000 automated research microscope (Leica, Dollard-des-Ormeaux, QC, Canada) equipped with a Lumenera InfinityX-21 digital camera using Infinity Capture software (Lumenera Corporation, Ottawa, ON, Canada) or analyzed by confocal microscopy using an Olympus Fluoview 1000 (FV1000) laser-scanning IX81-ZDC inverted microscope (Olympus Canada, Markham, ON, Canada). For the quantitative analysis of the number of Na_v_1.8-positive neurons, three immunofluorescence stained non-consecutive sections (90 μm apart from each other in the z axis) were imaged per ganglion. The data were collected from three animals at each time point (3, 8, and 14 days following CFA injections). Three age-matched sham rats were used as controls to set standard acquisition parameters (laser power, HV gain, offset). The threshold for negative cells was determined with MetaMorph (version 7.7 from Molecular Devices, LLC, Sunnyvale, CA, USA). All neurons showing a higher mean intensity than the baseline threshold were considered as Na_v_1.8-positive cells. To quantify the proportion of Na_v_1.8-positive cells within a defined subset of sensory neurons, we counted the number of positive neurons for Na_v_1.8 detected in NF200-immunoreactive neuronal profiles.

### Preparation of DRG neurons

Neurons were acutely dissociated from lumbar dorsal root ganglia of adult rats and maintained in a short-term primary culture to be used within a 20 h period, as previously described [32]. Briefly, L4-L6 DRGs isolated from sham and CFA-injected rats were freed from their adherent connective tissues. After washing with calcium-magnesium free PBS (pH 7.4), DRGs were incubated sequentially for 120 min in enzyme solutions containing collagenase A (1 mg/ml; Roche Diagnostics, Indianapolis, IN, USA) and then trypsin (0.25%; GIBCO, Burlington, ON, Canada). Subsequently, ganglia were mechanically dissociated into single cells by repeated trituration through a fine-polished Pasteur pipette in culture medium containing 1:1 Dulbecco’s modified Eagle’s medium (DMEM, Invitrogen) and Ham’s F12 supplemented with 10% fetal bovine serum (GIBCO) and 1% penicillin (100 U/ml)/streptomycin (0.1 mg/ml). Isolated neurons were gently centrifuged (50 *g* for 3 min), plated onto poly-D-lysine/laminin-coated glass coverslips, and maintained at 37°C in a humidified 95% air/5% CO_2_ incubator before they were used for in vitro patch-clamp electrophysiology and immunocytochemistry. The immunocytochemical detection of Na_v_1.8- and NF200-positive cells was performed 48 h after plating to allow them sufficient time to adhere. Isolated DRG neurons were then fixed for 15 min with 4% PFA before they were processed for immunostaining as described above.

### Electrophysiological measurements

Total sodium currents (INa) and TTX-R Na_v_1.8 currents were recorded from single, large-soma DRG neurons (Capacitance >70 pF) in the whole-cell patch-clamp configuration 14 to 20 h after plating, using an Axopatch 200 B amplifier (Molecular devices, Sunnyvale, CA, USA). No significant difference was found in the capacitance between any of the groups. Short-term culture provided cells with truncated (<10 μm) axonal processes that can be voltage clamped readily and reliably and minimized changes in electrical properties that can occur in long-term culture. All experiments were performed at room temperature (21 to 23°C).

The intracellular recording electrodes were fabricated from borosilicate glass capillary tubes (Warner Instrument, Hamden, CT USA), pulled using a two-step vertical micropipette puller P83 (Narishige, Japan) and heat-polished on a microforge (Narishige). For sodium current measurements, the pipette solution contained (in mM): 10 NaCl, 140 CsCl, 10 EGTA, 1 MgCl_2_, 2 Na2ATP, 10 HEPES; pH adjusted to 7.2 by CsOH. Osmolarity was adjusted to 300 mOsm/l with sucrose. Pipettes had a resistance of 2 to 4 MΩ when filled with the pipette solution. Capacity transients were cancelled using computer-controlled circuitry and series resistance was compensated (>85%) in all experiments. The external solution contained (in mM): 35 NaCl, 65 NMDG-Cl, 30 TEA-Cl, 0.1 CaCl_2_, 0.1 CoCl_2_, 5 MgCl_2_, 10 HEPES, and 10 glucose (pH adjusted at 7.4 by NaOH and osmolarity adjusted to 300 mOsm/l). The TEA-Cl and CoCl_2_ was used to inhibit endogenous K^+^ and Ca^2+^ currents, respectively. The sodium concentration was reduced to 35 mM in order to maintain an adequate clamp of the current. After formation of a tight seal, membrane resistance and capacitance were determined.

Total sodium currents were recorded with a 5-ms prepulse to −120 mV followed by a 500-ms test pulse. TTX-R Na_v_1.8 currents were isolated by prepulse inactivation as described earlier [33,34]. Briefly, standard current–voltage (I-V) families were constructed using a holding potential of −120 mV with 500-msec prepulses to −50 mV before each depolarization to inactivate the fast TTX-sensitive (TTX-S) currents. Thus, standard I-V curves were obtained by the application of a series of test pulses to voltages that ranged from −70 to +40 mV in 10 mV increments after the prepulse inactivation protocol. The voltage dependence of steady-state inactivation was measured by applying a double-pulse protocol consisting of a 500-ms conditioning potential (−120 to −10 mV, 5 mV increments) followed by a fixed test pulse (−10 mV, 50-ms). The current amplitude (I) was normalized to the maximum control current amplitude (Imax). For action potential measurements, the potassium channel blocker CsCl was replaced by an equimolar concentration of K-aspartate in the intracellular solution. The external solution contained (in mM): 145 NaCl, 1.8 CaCl_2_, 5.4 KCl, 2 MgCl_2_, 20 HEPES, and 10 glucose (pH adjusted at 7.4 by NaOH and osmolarity adjusted to 300 mOsm/l). Following formation of a gigaseal, a series of 1 ms current steps in 0.1 nA increments was injected into the cell under current-clamp mode. The threshold current needed to trigger an action potential was compared between control and CFA-treated animals in the presence or absence of TTX.

### Data analysis

The peak inward current values at each potential were plotted to generate I-V curves. Conductance (G) was determined as I/(Vm-Vrev), where I is the current, Vm is the potential at which current is evoked, and Vrev is the reversal potential of the current. Activation was fitted with the following Boltzman equation: G = Gmax/[1 + exp[(V_1/2_ – Vm)/k]], where Vm is the test pulse voltage potential at which current is evoked, Gmax is the calculated maximal conductance, V_½_ is the potential of half activation or inactivation, and k is the slope factor. The normalized curves were fitted using a Boltzmann distribution equation: I = Imax/[1 + exp[(V_1/2_ – Vm)/k]], where Imax is the peak sodium current elicited after the most hyperpolarized prepulse, Vm is the pre-conditioning pulse potential, V_1/2_ is the half maximal sodium current, and k is the slope factor. Sodium currents were recorded using a Digidata 1440 A data acquisition system (Molecular devices) digitized at 10 kHz, low-pass filtered at 2 kHz and captured using pClamp software (v10.2, Molecular devices). For current density measurements, the currents were divided by the cell capacitance as read from the amplifier. The offset potential was zeroed before patching the cells and leakage current was digitally subtracted online using hyperpolarizing potentials, applied after the test pulse. Curves were plotted and fitted using Origin software (OriginLab Corporation, Northampton, MA, USA).

### Statistics

Calculations and statistical analyses were performed using Prism 6.0 (Graph Pad Software, San Diego, CA, USA). All data are given as mean ± standard error of the mean (SEM). *P* values <0.05 were considered statistically significant. Von Frey, plethysmometer, and electrophysiological data as well as the immunostaining data comparing the proportion of Na_v_1.8-positive neurons in CFA-treated rats to sham animals were analyzed using one-way ANOVA followed by a Holm-Sidak post hoc test. qRT-PCR and Na_v_1.8 immunolabeling intensity in the sciatic nerve were compared between sham and CFA-treated rats with unpaired Student’s *t*-test.

## Results

### Changes in Na_v_1.8 channel distribution and expression during development of chronic inflammation

**Figure 1:**
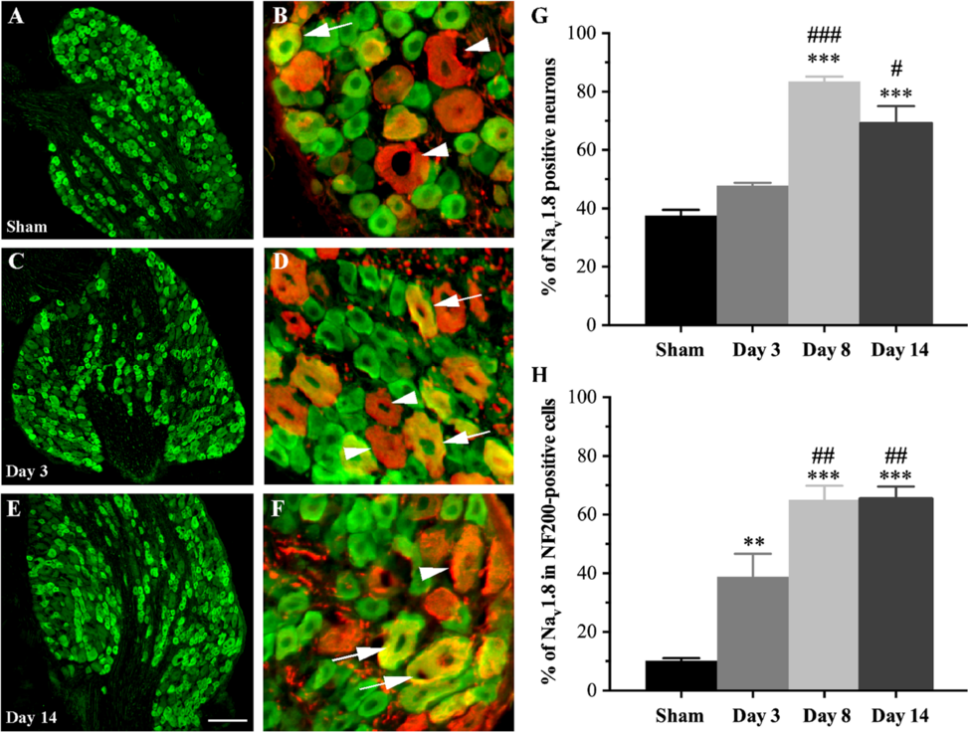
**Cellular distribution of Na**_**v**_**1.8 in rat DRG neurons. (A–F)** Immunohistochemical distribution of Na_v_1.8 in DRG neurons isolated from sham and CFA-treated rats. Panels show the localization of Na_v_1.8 (green) in NF200-positive neurons (red, arrowheads). Yellow signal indicates double-labeled neurons. After inflammation, numerous large NF200-positive ganglion cells co-express Na_v_1.8 (arrows). **(G)** Proportion of Na_v_1.8-immunoreactive neurons in lumbar DRGs. The number of neurons expressing Na_v_1.8 increases in CFA-inflamed rats compared to sham. **(H)** Percentage of NF200-containing DRG neurons expressing Na_v_1.8. The proportion of dually stained cells increases after CFA-induced chronic inflammation. Data in panels **G** and **H** shown as mean ± SEM. Asterisks denote a statistically significant increase as compared with sham (4 rats/group, 10 sections/rat; ***P <0.001 or **P <0.01; ANOVA followed by Sidak’s multiple comparisons test MCT)). ^#^Statistically different from day 3; ^###^*P* <0.001, ^##^*P* <0.01, ^#^*P* <0.05). Scale bars: 200 μm in **A, C,** and **E** and 20 μm in **B, D,** and **F**.

Pharmacological and physiological studies suggest that proinflammatory cytokines involved in the generation of pain sensitize primary afferent nociceptors by increasing voltage-gated Na^+^ currents [4,35]. In the present study, we therefore evaluated whether the cellular distribution of Na_v_1.8 within DRG neurons was altered by hind paw injection of CFA. To do so, we first determined, by immunohistochemical staining, the proportion of Na_v_1.8-expressing neurons in DRG tissues of sham and CFA-inflamed rats (Figure 1). Strong Na_v_1.8 labeling was evident in small to large ganglion cell bodies, this staining being completely prevented by preincubation with the cognate peptide (data not shown). Quantitative analysis revealed that approximately 35% of sensory neurons of all sizes displayed Na_v_1.8 immunoreactivity in sham animals (Figure 1A,G). The number of Na_v_1.8-positive cells significantly increased on days 8 (84% ± 1.6; ***P <0.001; Figure 1G) and 14 (69% ± 6; ***P <0.001; Figure 1E,G) following intraplantar administration of CFA, compared to sham (38% ± 2; Figure 1A,G). A time-related effect was also seen when comparing days 8 (^###^*P* <0.001) and 14 (^#^*P* <0.05) to the early day 3 (Figure 1G).

**Figure 2:**
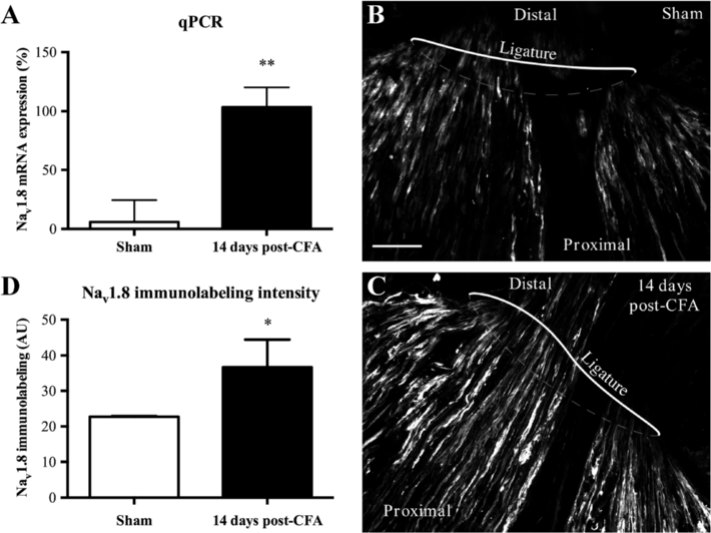
**Changes in the expression and distribution of Na**_**v**_**1.8 associated with CFA-induced inflammation in rats. (A)** Na_v_1.8 mRNA levels of the ipsilateral hind paw are determined by qRT-PCR for sham animals and 14 days following CFA injection. Data are expressed as mean ± SEM (6–8 rats/group). **P <0.01, CFA alone vs. sham (unpaired Student’s *t*-test). **(B, C)** Immunohistochemical staining of Na_v_1.8 channels in the rat sciatic nerve proximal to the lesion site 48 h after ligation. The ligature was placed around the sciatic nerve proximal to the trifurcation on day 12 post-CFA. Scale bar: 100 μm. **(D)** Accumulation of Na_v_1.8-like immunoreactivity is significantly increased in 14 day post-CFA rats compared to sham animals (*P <0.05; unpaired Student’s *t-*test).

To identify the subpopulation of large sensory neurons expressing Na_v_1.8, tissue sections of DRGs were then processed for double-labeling immunohistochemistry combining Na_v_1.8 antibodies with the high molecular weight (200 kDa) neurofilament protein NF200, a myelinated A-fiber ganglion cell marker. We found that about 10% of NF200-immunopositive cells expressed Na_v_1.8-like immunoreactivity in sham rats (Figure 1B, H). In contrast, NF200 co-localized extensively with Na_v_1.8 during the persistent inflammatory state. Specifically, 39% (± 8; **P <0.01), 65% (± 5; ***P <0.001), and 65% (± 4; ***P <0.001) of NF200-positive ganglion cells exhibited Na_v_1.8-like staining at days 3, 8, and 14 after CFA injection, respectively (Figure 1D,F,H). Secondly, there were also significant differences between days after intraplantar CFA injection (Figure 1H). This increase in Na_v_1.8 immunolabeling was accompanied by marked changes in its mRNA expression (Figure 2A). Compared to sham rats, high levels of Na_v_1.8 transcripts were expressed in ipsilateral lumbar DRGs isolated 14 days post-CFA.

### Intra-axonal transport of Na_v_1.8 channels increases under persistent inflammation

We next examined if the gradual increase of the ipsilateral Na_v_1.8 immunofluorescence and mRNA expression in DRG cell bodies observed after CFA-induced inflammation was followed by axonal protein trafficking toward sensory afferent terminals in peripheral tissues. To this end, the sciatic nerve was ligated for 2 days to verify the accumulation of transported Na_v_1.8 at the ligation site. In sham animals, we found, using immunofluorescence staining, that Na_v_1.8 was accumulating in the portion of the ligated sciatic nerve that was proximal to the lumbar DRG (Figure 2B). This indicates that Na_v_1.8 was anterogradely transported along the sciatic nerve to reach the peripheral terminals. No accumulation of Na_v_1.8 was detected on the distal site and on the non-ligated contralateral side (not shown). More importantly, we found an increase in Na_v_1.8-like immunoreactivity in the sciatic nerve at the proximal side, 14 days after CFA injection, compared to sham (*P <0.05; Figure 2C,D). These results thus suggest that newly synthesized Na_v_1.8 proteins can be redistributed to peripheral afferent terminals after chronic tissue inflammation. Alternatively, the accumulation of Na_v_1.8 channels at the ligation site may rely on mRNA axonal transport and local protein synthesis.

### CFA-induced modulation of total I_Na_ currents in large-diameter sensory neurons

**Figure 3:**
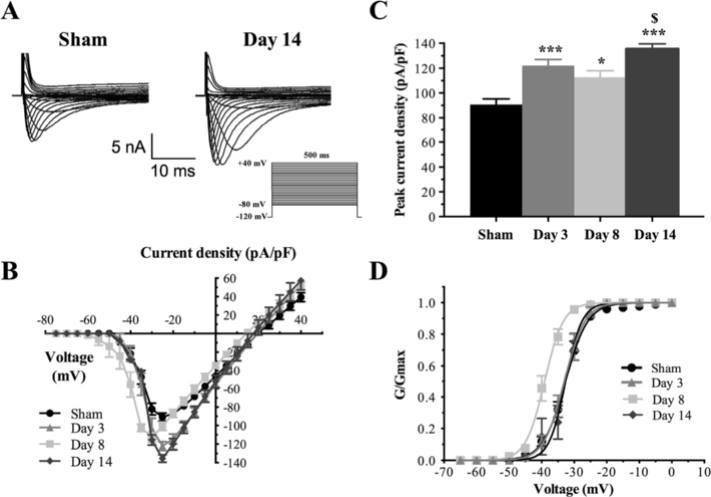
**Total I**_**Na **_**currents in large sensory neurons following exposure to CFA. (A)** Whole-cell voltage-clamp current traces of I_Na_ are recorded from sham and CFA large-soma DRG neurons. Total currents were elicited by a series of 500-ms test pulses ranging from –80 to +40 mV in 5 mV steps. **(B)** I-V curves of total I_Na_ currents obtained from large-soma rat DRG neurons (Capacitance >70 pF). Maximum peak currents are observed at –25 mV in all groups with the exception of day 8 post-inflammation (–30 mV). **(C)** Histogram showing that the development of chronic inflammation induced by CFA intraplantar injection (days 3, 8, and 14 post-CFA) increases total I_Na_ peak currents. Data shown as mean ± SEM (*P <0.05, ***P <0.001 vs. sham; ^$^*P* <0.05 vs. day 8; ANOVA followed by Sidak’s MCT; n = 8–11). **(D)** Voltage-dependent activation of total I_Na_ currents in large sensory neurons from inflamed rats. At day 8, CFA shifts the activation curve in a hyperpolarizing direction (V_1/2act_ = –39.7 ± 1.6 mV for inflamed rats vs. –32.7 ± 0.8 mV for sham group; **P <0.01). Half-activation and half-inactivation potentials and slope factors are summarized in Additional file 2: Table S2.

To determine if the changes in Na_v_1.8 expression altered the total sodium current (I_Na_) during development of chronic inflammation, we first measured the density of I_Na_ using a whole-cell configuration of the patch-clamp technique and characterized its kinetic properties in both normal and inflamed large sensory neurons. Representative recordings of total I_Na_ currents from large-soma rat DRG neurons (Capacitance >70 pF) are shown in Figure 3A. A series of depolarizing voltage commands from −80 to + 40 mV were applied to activate all sodium channels in the cells. Maximum I_Na_ density increased at day 3 (−122.1 ± 4.9 pA/pF; ***P <0.001), day 8 (−112.8 ± 5.2 pA/pF; *P <0.05), and day 14 (−135.4 ± 4.1 pA/pF; ***P <0.001) in DRG neurons as CFA-induced inflammation developed, compared to sham cells (−90.7 ± 4.6 pA/pF) (Figure 3B,C; Additional file 2: Table S2). I_Na_ density was substantially higher on day 14 post-CFA, when compared to day 8 (^$^*P* <0.05). We next examined the voltage-dependence of activation of I_Na_ in CFA-treated and sham large DRG neurons. CFA treatment shifted the half-activation potential (V_1/2act_) to a more negative potential in large-soma neurons isolated from rats treated for 8 days with CFA (−39.7 ± 1.6 mV; **P <0.01) compared to sham neurons (−32.7 ± 0.8 mV), but had no significant effects in animals treated for 3 or 14 days (Figure 3D; Additional file 2: Table S2). These results therefore suggest that CFA enhanced or induced expression of new sodium channels in large DRG neurons. We next sought to determine if a change in the contribution of Na_V_1.8 could be involved.

### CFA increases Na_v_1.8 currents in large-diameter sensory neurons

**Figure 4:**
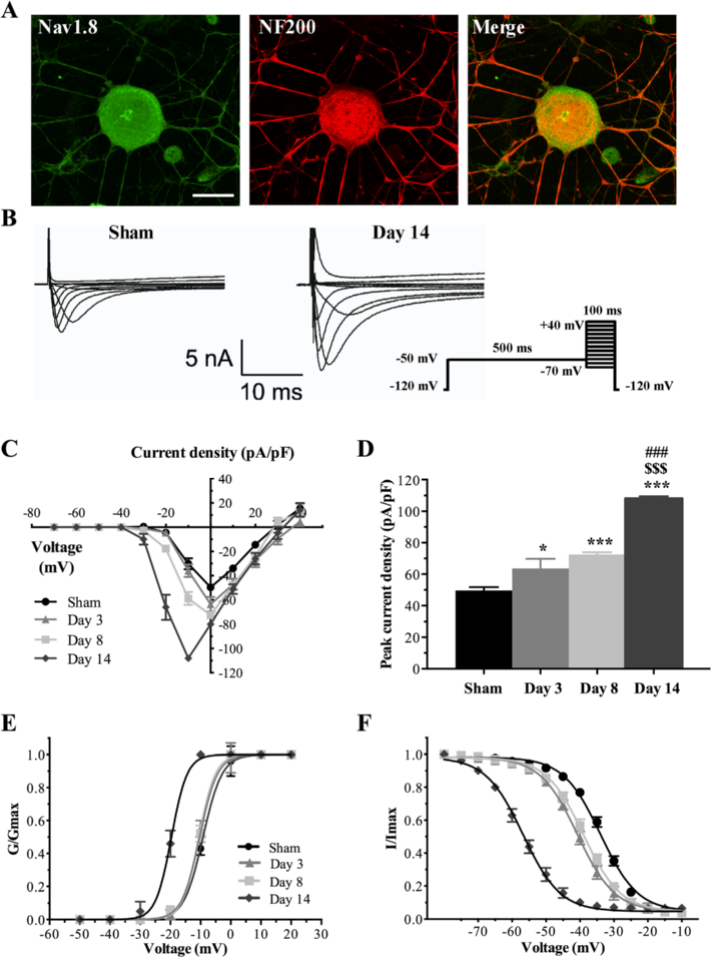
**Na**_**v**_**1.8 currents are enhanced in large-sized DRG neurons from inflamed rats. (A)** Immunofluorescence staining of Na_v_1.8 (green) and NF200 (red) on acutely dissociated primary afferent neurons, 14 days post-CFA. Merge images show dually labeled large-sized sensory neurons (yellow). **(B)** Isolation of TTX-resistant Na_v_1.8 currents in large-sized sensory neurons from sham and inflamed rats. Na_v_1.8 currents are significantly increased post-CFA. Representative I-V curves of currents are determined using the pulse protocol indicated in the inset. **(C)** I-V curves of Na_v_1.8 currents obtained from large-soma DRG neurons. The peak maximum current is observed at 0 mV in all groups, with the exception of day 14 (–10 mV). **(D)** Peak Na_v_1.8 current densities are significantly increased at days 3, 8, and 14 post-CFA injection (***P <0.001 *P <0.05 vs. sham; ^$$$^*P* <0.001 vs. day 3; ^###^*P* <0.001 vs. day 8; ANOVA followed by Sidak’s MCT; n = 6–13). **(E, F)** Kinetic properties of Na_v_1.8 currents in large-sized sensory neurons. CFA treatment induces a leftward shift of the activation **(E)** and inactivation **(F)** curves of Na_v_1.8 current. Half-activation and half-inactivation potentials and slope factors are summarized in Additional file 3: Table S3.

Dual immunostaining revealed that Na_v_1.8 and NF200 immunoreactivities co-localized extensively over large-sized sensory neurons in acutely dissociated DRG cell cultures isolated from CFA-treated rats (Figure 4A). We then tested if some of the changes in the biophysical properties of total I_Na_ correlated with an enhanced expression of Na_v_1.8 in acutely dissociated lumbar NF200-positive sensory neurons (Capacitance >70 pF). In order to isolate the contribution of the TTX-R Na_v_1.8 current from TTX-S sodium channels, we used a 500 ms inactivation prepulse to −50 mV. This protocol ensured that fast inactivating TTX-S channels did not contribute to I_Na_ measurements during the test pulse and biophysically isolate the more slowly inactivating TTX-R currents [33,34,36]. Furthermore, in this set of experiments, the membrane potential was held at −70 mV to inhibit the potential contribution of Na_v_1.9 channels, thus leaving solely the Na_v_1.8 current to be measured [37,38]. The resulting Na_v_1.8 currents were elicited by applying series of 100 ms test pulses between −70 and +40 mV in 10 mV increments (Figure 4B).

Representative recordings of Na_v_1.8 currents from both sham and inflamed large sensory neurons are shown in Figure 4B. Consistent with our immunostaining experiments, we found that CFA significantly increased Na_v_1.8 current density compared to sham DRG neurons. I-V analysis revealed that CFA significantly increased the average maximum current density at days 3 (−63.7 ± 6.0 pA/pF; *P <0.05), 8 (−72.6 ± 2.9 pA/pF; ***P <0.001), and 14 (−108.1 ± 1.5 pA/pF; ***P <0.001) compared to sham DRG neurons (−49.6 ± 2.4 pA/pF) (Figure 4C,D). Interestingly, maximum current density at day 14 increased by more than 30% at day 14 compared to days 3 (^$$$^*P* <0.001) and 8 (^###^*P* <0.001) (Figure 4D; Additional file 3: Table S3).

Because Na_v_1.8 channel kinetics differs from TTX-S channels it may influence the threshold for triggering action potentials, modulate the transmission of the neuronal electrical impulse and, as a consequence, influence nociception. We therefore determined if the development of chronic inflammation altered the voltage-dependence of activation and inactivation (availability) of Na_v_1.8 currents. Our results show that CFA significantly hyperpolarized the midpoint of activation (V_1/2act_) in large neurons from ipsilateral DRG after 14 days (−20.25 ± 0.6 mV) compared to sham neurons (−8.79 ± 1.01 mV; ***P <0.001). Surprisingly, no shift in the activation curve was observed at days 3 (−9.3 ± 0.23 mV, n = 6; ^$$$^*P* <0.001) and 8 (−12.26 ± 1.01 mV, n = 7; ^###^*P* <0.001) after CFA injection (Figure 4E, Additional file 3: Table S3).

CFA administration shifted the availability of Na_v_1.8 channels (steady-state inactivation) in the hyperpolarizing direction at all time-points (Figure 4F) with half-inactivation potentials (V_1/2inact_) of −39.9 ± 1.34 mV (**P <0.01), −38.4 ± 1.1 mV (*P <0.05), and −56.3 ± 1.3 mV (***P <0.001) at days 3, 8, and 14, respectively, compared to control conditions (−33.6 ± 0.3 mV). Significant differences in V_1/2inact_ were also observed between day 14 and days 3 (^$$$^*P* <0.001) and 8 (^###^*P* <0.001). Slope factors, k_act_ and k_inact_, remained unchanged between sham and CFA-treated groups (Additional file 3: Table S3). Despite a significant shift in Na_v_1.8 steady-state inactivation induced by CFA, examination of Figure 4F reveals that the loss of channel availability will be in order of 20% for a resting membrane potential around −70 mV. These results indicate that the drastic augmentation of 120% from −49.6 ± 2.4 pA/pF to −108.1 ± 1.5 pA/pF (Figure 4C,D) in Na_v_1.8 current density is not due to changes in the availability of the channels but more likely result from enhanced expression of Na_v_1.8 channels that largely compensate for the loss of channels to inactivation, thus confirming the qPCR and immunostaining data.

### CFA increases excitability in large-diameter sensory neurons

**Figure 5:**
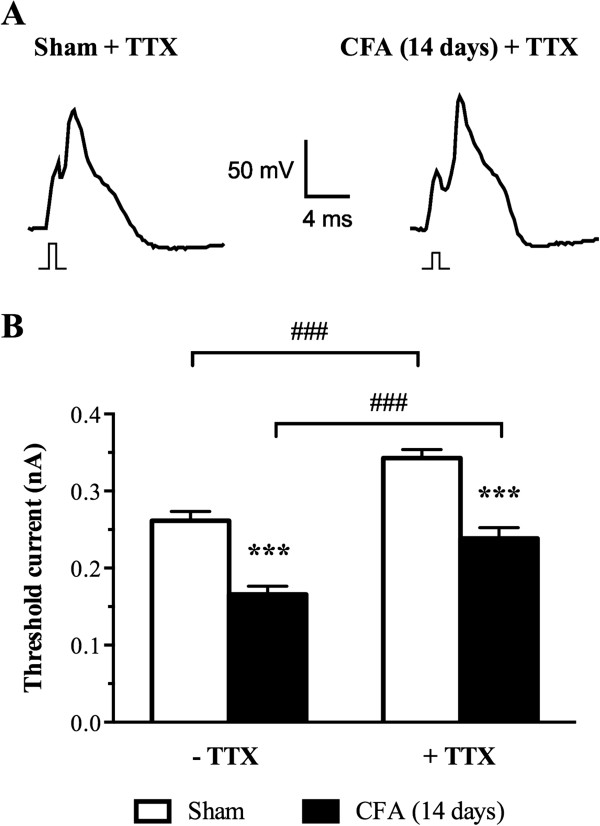
**CFA increases excitability in large sensory neurons. (A)** Representative recordings of DRG action potentials (AP) from sham and CFA-treated rats in the presence of 100 nM of tetrodotoxin (TTX). APs were triggered by a 1 ms current stimulus. The minimal (threshold) current amplitude needed to trigger APs was smaller in neurons from CFA-treated animals as illustrated by the smaller amplitude stimulus shown under each action potential. **(B)** Average threshold currents in sham and CFA conditions. CFA significantly decreased the amplitude of the current needed to trigger an action potential in neuronal cells exposed or not to 100 nM of TTX. Data shown as mean ± SEM (***P <0.001, CFA vs. sham; ^###^*P* <0.001, –TTX vs. + TTX ANOVA followed by Sidak’s MCT; n = 14–22 from 3 rats in each condition).

The voltage dependence of activation of I_Na_ is known to determine the voltage threshold for triggering action potential in excitable cells. Our observation of a negative shift in the mid-activation potential of I_Na_ (Figure 4) suggests that the threshold potential for triggering an action potential is closer to the resting membrane potential and therefore renders CFA-treated DRG neurons more readily excitable. To test this hypothesis, we measured the current threshold (I_Th_) needed to trigger an action potential under current clamps in large sensory neurons isolated from sham or CFA-treated rats. Figure 5 shows that CFA significantly reduced I_Th_ from 0.26 ± 0.01 nA in sham cells to 0.17 ± 0.01 nA in CFA-treated large sensory neurons (***P <0.001). To test for a specific contribution of TTX-R channels, we applied TTX in concentrations known to completely block TTX-S channels. Following application of 100 nM TTX, the action potential threshold was increased to 0.24 ± 0.01 nA and 0.34 ± 0.01 nA in CFA- and sham-treated neurons, respectively (Figure 5A,B). As expected, from the blockade of TTX-sensitive current, application of TTX increased the current needed to trigger an action potential by 31% and 42% in sham- and CFA-treated neurons, respectively. However, the changes in I_Th_ between CFA- and sham-treated cells slightly decreased from 35% in control to 30% upon application of TTX. These results therefore indicate that the major changes in I_Th_ are due to a contribution of TTX-R channels with a smaller contribution of 5% coming from TTX-S and therefore reinforce the idea that inflammation increases large sensory neuron excitability by recruiting Na_v_1.8 channel.

### Ambroxol blocks CFA-induced potentiation of Na_v_1.8 currents in large-diameter sensory neurons

**Figure 6:**
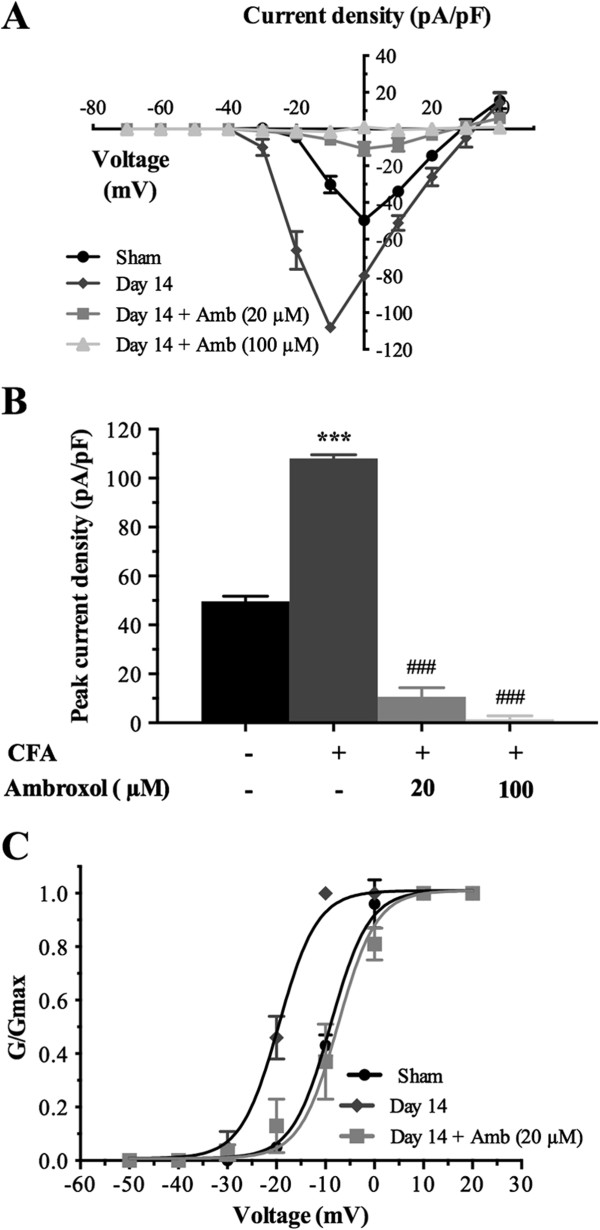
**Ambroxol treatment blocks the changes in the biophysical properties of Na**_**v**_**1.8 in large-sized DRG neurons extracted from rats 14 days post-CFA. (A)** I-V curves of Na_v_1.8 currents obtained from sham and inflamed large sensory neurons. **(B)** Histogram showing the effects of different concentrations of ambroxol (20 and 100 μM) on the increased Na_v_1.8 peak current induced by CFA (***P <0.001, inflamed vs. sham large sensory neurons; ^###^*P* <0.001 compared to inflamed DRG neurons; ANOVA followed by Sidak’s MCT; n = 6–13). **(C)** Ambroxol also significantly inhibits the leftward shift of the activation curves of Na_v_1.8 current. Half-activation potential and slope factor are summarized in Additional file 3: Table S3. Note that the data corresponding to sham and day 14 after CFA treatment are the same as the ones in Figure 4.

To further confirm a contribution of Na_v_1.8 to I_Na_ during chronic inflammation, we measured the sodium current in isolated large sensory neurons from CFA-injected rats following application of ambroxol, a preferring blocker of Na_v_1.8. In agreement with our previous results, I-V analysis revealed that the increase in I_Na_ was considerably reduced following acute application of ambroxol on neurons 14 days post-CFA (Figure 6). The average maximum current amplitude was decreased by 90% in inflamed large sensory neurons, following 30 min pre-incubation with 20 μM ambroxol (−10.7 ± 3.7 pA/pF vs. –108.1 ± 1.5 pA/pF; ^###^*P* <0.001) (Figure 6A,B). At the highest concentration tested (100 μM), the Na_v_1.8-preferring sodium channel blocker completely prevented the increase in peak current density induced by CFA (−1.5 ± 1.4 pA/pF vs. –108.1 ± 1.5 pA/pF; ^###^*P* <0.001) (Figure 6A,B).

Our results also showed that ambroxol (20 μM) significantly blocked the hyperpolarizing shift in the activation curve of I_Na_ observed in response to CFA (Figure 6C). The half-activation potential (V_1/2act_) was shifted to −8.52 ± 3.39 mV from that of CFA control condition (−20.25 ± 0.63 mV) after 20 μM ambroxol treatment (^###^*P* <0.001; Additional file 3: Table S3), thus reinforcing the notion that enhanced expression of Na_v_1.8 increases the excitability of inflamed neurons.

### Inhibition of Na_v_1.8 channel function by ambroxol

**Figure 7:**
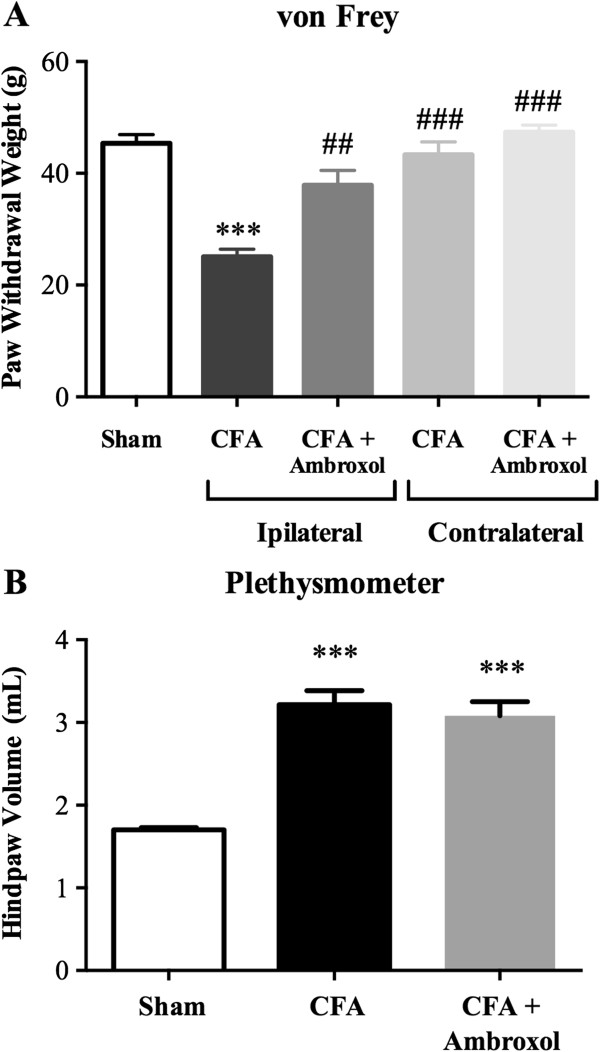
**Effect of ambroxol on CFA-induced mechanical allodynia and edema.** Mechanical sensitivity **(A)** and volume **(B)** of the ipsilateral hind paw are determined for sham animals and 14 days following CFA injection. Effects of ambroxol (0.3 mg/kg cumulative dose) were determined 1 h following administration of the last of 3 intrathecal injections (0.1 mg/kg injected on days 3, 8, and 14) on day 14 post-CFA. Data are expressed as mean ± SEM (6–8 rats/group). ***P <0.001, compared to sham. ^##^*P* <0.01, ^###^*P* <0.001 vs. CFA ipsilateral alone; One-way ANOVA followed by Sidak’s post-hoc comparisons test.

We then determined the ability of i.t. injections of ambroxol, a Na_v_1.8-preferring Na^+^ channel blocker, to block the signs of nociception induced 14 days post-CFA. As shown in Figure 7A, CFA-treated rats exhibited paw withdrawals at reduced mechanical forces (25 g ± 1.4) compared to sham animals (45 g ± 1.6; ***P <0.001), consistent with the development of mechanical hypersensitivity. In addition, CFA injection caused a marked increase in paw volume indicative of edema development, compared to shams (3.2 ml ± 0.2 vs. 1.7 ml ± 0.1, respectively; ***P <0.001; Figure 7B). Interestingly, i.t. ambroxol (0.1 mg/kg acute dose, 0.3 mg/kg cumulative dose) reduced mechanical allodynia by 63% in the CFA model of inflammatory pain (^##^*P* <0.01; Figure 7A). Additionally, ambroxol had no effect on paw withdrawal threshold of the contralateral non-inflamed paw, revealing specific anti-allodynic effects of the drug (Figure 7A). Furthermore, Na_v_1.8 channel blockade by i.t. ambroxol did not modify the inflammation-induced paw swelling (Figure 7B).

## Discussion

Voltage-gated sodium channels play a fundamental role in pain sensation under both physiological and pathological conditions [4,5]. Among them, Na_v_1.8 has been shown to regulate sensory neuron excitability and thus to participate in the peripheral sensitization associated with the development of chronic pain [4,6]. Our findings herein revealed that Na_v_1.8 is up-regulated in large myelinated Aβ-fiber neurons and is subsequently anterogradely transported from the DRG cell bodies along the axons toward the periphery after CFA-induced inflammation. We also demonstrated that the development of peripheral chronic inflammation was associated with both enhanced I_Na_ and Na_v_1.8 current densities in NF200-positive Aβ-fiber neurons. Persistent inflammation further leads to time-dependent changes in Aβ-fiber neuron excitability by shifting the voltage-dependent activation and steady-state inactivation curves of Na_v_1.8 in the hyperpolarizing direction. In good agreement, we also found, using a current-clamp mode, that CFA-induced persistent inflammation enhances the excitability of Aβ-fiber neurons. Finally, we found that ambroxol, a Na_v_1.8-preferring blocker, significantly reduces the potentiation of Na_v_1.8 currents observed following intraplantar CFA injection and concomitantly blocks CFA-induced mechanical allodynia.

### Na_v_1.8 is up-regulated in Aβ-fiber neurons under persistent inflammation

Functional alterations in the properties of Aβ-fiber neurons may account for the increased pain sensitivity observed under peripheral chronic inflammation [2]. In particular, there is now considerable evidence that Aβ afferent fiber neurons undergo significant changes in their electrical characteristics under chronic pain conditions [39-42]. We hypothesized here that the alteration in Na_v_1.8 sodium channel expression, trafficking, and activity might substantially contribute to the enhanced excitability in injured Aβ sensory neurons, thus allowing the development of mechanical allodynia. In the present study, we first examined the expression of Na_v_1.8 in the defined subpopulation of NF200-positive neurons over a 14-day period following CFA injection. The immunocolocalization experiments revealed that Na_v_1.8 is present in about 10% of NF200-positive large-soma DRG neurons from sham animals. These results are in accordance with previous studies reporting that 10% to 30% of large-diameter sensory neurons contain Na_v_1.8 mRNA or are immunohistochemically labeled for the Na_v_1.8 protein in sham animals [13-15,17-24,43,44]. Likewise, Shields et al. recently found, using Na_v_1.8-cre mice, that an even higher percentage of NF200-labeled neurons (around 40%) was Na_v_1.8-cre-positive [25].

Our results further demonstrated that Na_v_1.8 expression increases dramatically in DRG neurons during inflammation, with 69% of all sensory neurons and 65% of NF200-positive ganglion cells exhibiting Na_v_1.8 immunostaining 14 days post-CFA (compared to 35% and 10% in sham animals, respectively). This up-regulation of Na_v_1.8 expression in DRG tissue has also been highlighted by others following short-term exposure to carrageenan or CFA (one to four days post-insult) [16,20,45,46]. The long-term effects of proinflammatory agents on Na_v_1.8 expression was only observed 14 days after repeated intraplantar injections of PGE_2_ or over a 28-day time course following intra-articular injection of CFA [47-49]. However, to date, relatively few studies have examined how these inflammatory conditions specifically affect the expression level of Na_v_1.8 in injured Aβ sensory neurons. Consistent with our findings, Coggeshall et al. have observed significant changes in Na_v_1.8 expression (6-fold increase), reaching close to 60% of labeled myelinated axons in digital nerves 48 h post-CFA [50]. Altogether, these findings suggest that up-regulation of Na_v_1.8 under peripheral chronic inflammation may be responsible for at least some of the changes in Aβ-fibers.

### Persistent peripheral inflammation increases the anterograde transport of Na_v_1.8

Redistribution of voltage-gated channels along the peripheral axolemma has also been proposed to significantly influence nociceptor excitability and to be in part responsible for the spontaneous ectopic discharges generated by damaged peripheral sensory nerves [4,5,51]. In this study, we thus asked whether Na_v_1.8 was transported to injured peripheral terminals during chronic tissue inflammation. Our results revealed that Na_v_1.8 immunoreactivity was increased in axons proximal to the site of injury in uninflamed rat sciatic nerve 48 h following ligation. More importantly, CFA-induced peripheral inflammation resulted in a significantly increased accumulation of Na_v_1.8 immunostaining at the ligated site, compared to uninflamed nerves. These data thus indicate that non-physiological nociceptive afferent excitation induces intra-axonal anterograde trafficking of Na_v_1.8 from DRG cell bodies to peripheral terminals. In support of these results, rats that received CFA into the upper lip/whisker pad were shown to exhibit an increase in Na_v_1.8 immunoreactivity in the infraorbital nerve 48 hours and one week after CFA treatment, revealing transport of Na_v_1.8 protein to the periphery [52].

Although the role of Na_v_1.8 in the generation and maintenance of neuropathic pain is not completely defined and remains controversial, it has been found in several nerve injury-based rodent models of peripheral neuropathy that Na_v_1.8 immunoreactivity strongly increased in the nerve proximal to the injury site [43,53,54]. Consistent with these findings, patients dealing with chronic neuropathic pain show increased Na_v_1.8 channel accumulation at the sites of nerve injury [55-59]. The massive redistribution in Na_v_1.8 immunoreactivity along the sciatic nerve might be attributed to the up-regulation of Na_v_1.8 mRNA and protein levels within the cell bodies of sensory neurons, as we observed 14 days post-CFA administration. We might also hypothesize that Na_v_1.8 mRNA redistribution in both myelinated and unmyelinated axons followed by local translation contributes to the increased immunoreactivity in the injured nerve, as previously reported in peripheral neuropathy [54,60]. These results reveal that the trafficking of Na_v_1.8 to sensory nerve endings, such as those found in the skin, may play a significant role in the development and maintenance of peripheral sensitization and chronic pain states. This also suggests that blocking Na_v_1.8 axonal transport may be effective for treating chronic neuropathic or inflammatory pain.

### Peripheral inflammation enhances total I_Na_ and Na_v_1.8 currents in Aβ-fiber neurons

Primary hyperalgesia is mainly due to sensitization of the peripheral terminals of nociceptors and is the consequence of an increase in their membrane excitability and/or a reduction in their action potential threshold. This alteration of the sensory transmission is typically triggered by pro-inflammatory mediators released by damaged tissues and infiltrating immune cells. Among these hyperalgesic agents, the prostaglandins PGE2 and PGD2 and the chemokine CCL2, as well as adenosine, serotonin, and substance P were found to regulate current amplitude and conductance of TTX-R sodium channels on small- and medium-sized sensory neurons [7,9,10,37,61,62]. However, to date, very few studies have investigated the changes in voltage-sensitive sodium currents in experimental models of persistent inflammation, and all of them have focused only on small/medium-sized primary afferent neurons [20,46,63-65]. Since allodynia is believed to be mediated by large Aβ-fibers, we investigated here whether the induction of chronic peripheral inflammation by an intraplantar injection of CFA induced significant changes in the biophysical properties of sodium channels (notably Na_v_1.8) in NF200-positive large Aβ-fiber neurons.

Our electrophysiological recordings performed in the whole-cell patch-clamp configuration revealed that both I_Na_ and Na_v_1.8 peak current densities were larger in the CFA-injected group at all time-points (3, 8, and 14 days) after CFA injection than in the sham group. Accordingly, we also found under current-clamp experiments that CFA-induced persistent inflammation decreased the current threshold for action potential initiation, thus enhancing the excitability of Aβ-fiber neurons. Our data are consistent with previous studies performed on small sensory neurons demonstrating that short-term exposure (<5 days) to carrageenan or CFA treatment produced an increase of both TTX-R and TTX-S Na^+^ current amplitudes [20,46,63-65]. However, as opposed to those studies, we demonstrated here that the increased amplitude was accompanied by hyperpolarizing shifts in the voltage-dependence of activation and steady-state inactivation. These findings thus suggest that the voltage-dependent and kinetic properties of DRG sodium currents can depend either on the neuronal cell-type (small vs. large sensory neurons), on the type of inflammatory agents used (carrageenan, CFA), or on the time course of inflammation (short- or long-term exposure).

Finally, our results revealed that the gating properties of I_Na_ and Na_v_1.8 sodium currents along with the up-regulation of Na_v_1.8 expression in DRG tissue are differentially regulated over the time course of the CFA challenge. These differences can be explained in part by the evolution of the early inflammation state to a transient inflammation condition at day 8 post-CFA, and finally to signs of arthritis at later time points (14 days) [66]. Furthermore, we also found that peripheral inflammation produced a leftward shift in activation voltage-dependency for the total sodium current at day 8 without any shift in the voltage-dependence of activation of Na_v_1.8. One possibility is that TTX-S currents account for the transient negative shift in activation voltage-dependency observed at day 8 post-CFA. Based on previous findings indicating that TTX-R Na_v_1.9 sodium currents are up-regulated in large sensory neurons in painful diabetic neuropathy [18], we might also hypothesize that this channel is possibly involved in the changes of voltage-dependent properties of I_Na_ in Aβ-fiber neurons, 8 days following induction of peripheral inflammation.

### Ambroxol blocks CFA-induced mechanical allodynia and potentiation of Na_v_1.8 currents in Aβ-fiber neurons

Several lines of behavioral evidence indicate that the Na_v_1.8 channel is an important contributor to the development of the hypersensitivity state that underlies inflammatory pain [4,5,11,12]. Indeed, studies with Na_v_1.8-null mice, Na_v_1.8 blockers, antisense technology, and short inhibitory RNA (siRNA)-mediated knockdown approaches have demonstrated that inhibition of Na_v_1.8 reduces the mechanical, thermal, and visceral responses in animal models of inflammatory pain [13,48,63,67-76]. In the present study, we showed that mechanical allodynia was significantly reduced at 14 days post-CFA by repeated i.t. administration of ambroxol (0.3 mg/kg cumulative dose), a Na_v_1.8-preferring channel inhibitor. Accordingly, ambroxol was also effective in reversing CFA-induced Na_v_1.8 current amplitudes and gating properties in Aβ-fiber neurons. These results are in accordance with previous studies demonstrating that ambroxol suppresses the nociceptive behaviors associated with the development of chronic painful conditions, including neuropathic and inflammation [27,29,77,78]. In good agreement with these findings, systemic or i.t. administration of Na_v_1.8 selective blockers (e.g., A-803467) was found to be active in reducing various nociceptive symptoms and to potently inhibit evoked and spontaneous firing of dorsal horn wide dynamic range neurons in inflammatory and neuropathic pain models [28,70,74,79,80]. Collectively, our findings suggest an interesting paradigm by which enhanced expression of Na_v_1.8 increases the amplitude of the action potentials and may promote a more rapid transmission of the electrical impulse and, by virtue of Na_v_1.8 biophysical properties, decrease the threshold for triggering action potentials and therefore increase excitability in Aβ-fiber neurons and its contribution to the development of mechanical allodynia under persistent inflammation.

### How can these changes in Na_v_1.8 function in large diameter myelinated Aβ-fiber neurons drive pain?

It is widely accepted that sensitization of C– and δ-fibers can directly result in increased pain. However, it is less obvious to explain how the increase of Na_v_1.8 function in myelinated Aβ-fibers, which normally convey low threshold touch, can fill some of the roles of C– and δ-fibers under persistent inflammation and lead to the recruitment of postsynaptic pain signaling pathways. Different mechanisms can be put forward to explain how Aβ afferent fibers might come to evoke tactile allodynia in the event of persistent inflammation. Notably, it has been proposed that large myelinated tactile Aβ afferent fibers, which normally arborize in laminae III–V, may sprout dorsally into the superficial laminae of the spinal cord dorsal horn and gain access to second order nociceptive neurons [81-87]. Alternatively, we can consider the possibility that the thick Aβ afferent fibers are the principal driver of pain sensation (tactile allodynia). Both peripheral nerve injury and chronic peripheral inflammation are known to potentiate intrinsic oscillations in membrane potential and increase ectopic discharges in DRG neurons [88,89]. Here, we found that CFA induced overexpression of Na_v_1.8 combined with a negative voltage shift in I_Na_ activation lowered the threshold for triggering action potentials. Therefore, one expects the enhanced oscillatory behavior to increase ectopic firing as the oscillations more frequently reach the action potential threshold. Crosstalk or ephapsis do not normally occur within the DRG, as each sensory neuron is isolated from the others [90,91]. However, chemically mediated cross-excitation in the DRG and transient cross-depolarization in neighboring cells were observed in 90% of neurons within the DRG following tetanic stimulation [90,92]. A similar mechanism may also explain the mechanical allodynia in our CFA model. As overexpression of Na_V_1.8 enhances excitability and the probability of spontaneous action potentials in DRG neurons, cross-depolarizations sufficient to evoke ectopic firing in neighboring nociceptive neurons may induce mechanical allodynia. Such a mechanism is supported by evidence of cross-excitation between A- and C-fibers [93].

## Abbreviations

CFA: Complete Freund’s adjuvant; DRG: Dorsal root ganglia; I_Na_: Total sodium current; I-V: Standard current–voltage; TTX-R: Tetrodotoxin-resistant; TTX-S: Tetrodotoxin-sensitive.

## Competing interests

The authors declare that they have no competing interests.

## Authors’ contributions

MBe performed the electrophysiological and immunohistochemical experiments presented in this manuscript. MAD realized the in vivo behavioral studies as well as the quantitative real-time PCR experiments. PT was involved in the immunostaining studies. MBi performed the electrophysiological studies under current-clamp mode. PS conceptualized the project and wrote the manuscript. NB, AC, RD, and SMP participated in the design of the experiments and edited the manuscript. All authors read and approved the manuscript.

## Authors’ information

Ahmed Chraibi, Stéphane Mélik-Parsadaniantz, and Philippe Sarret: co direction of the work.

## Supplementary Material

Additional file 1: Table S1 DNA oligonucleotides and probes used in the qPCR assay. Click here for file

Additional file 2: Table S2 Properties of I_Na_ currents in sham and inflamed large sensory neurons. V_1/2act_ is the membrane potential for half-maximal channel activation. k_act_ represents the slope factor for activation. *P <0.05, **P <0.01, and ***P <0.001 indicate statistically significant differences with sham group. ^$^*P* <0.05 compared to large-sized DRG neurons extracted from rats 14 days post-CFA to day 8. Numbers in parentheses reflect numbers of recorded neurons. Click here for file

Additional file 3: Table S3 Mean peak sodium current amplitude, activation, and steady-state inactivation characteristics of Na_v_1.8 currents in sham and inflamed large sensory neurons. V_1/2act_ and V_1/2inact_ are the membrane potentials for half-maximal channel activation or inactivation, respectively. k_act_ and k_inact_ represent the slope factors for activation and inactivation. *P <0.05, **P <0.01, and ***P <0.001 indicate statistically significant differences with sham group. ^$$$^*P* <0.001 and ^###^*P* <0.001 compared to large-sized DRG neurons extracted from rats 14 days post-CFA to days 3 and 8, respectively. Numbers in parentheses reflect numbers of recorded neurons. Ambroxol also significantly inhibits the leftward shift of the activation curves of Na_v_1.8 current (^###^*P* <0.001 compared to CFA-treated neurons). Click here for file
