# Supplementary figures and images for: Functional up-regulation of Nav1.8 sodium channel in Aβ afferent fibers subjected to chronic peripheral inflammation
Source: J Neuroinflammation. 2014 Mar 7;11:45. doi: 10.1186/1742-2094-11-45 (PMC4007624; doi:10.1186/1742-2094-11-45)

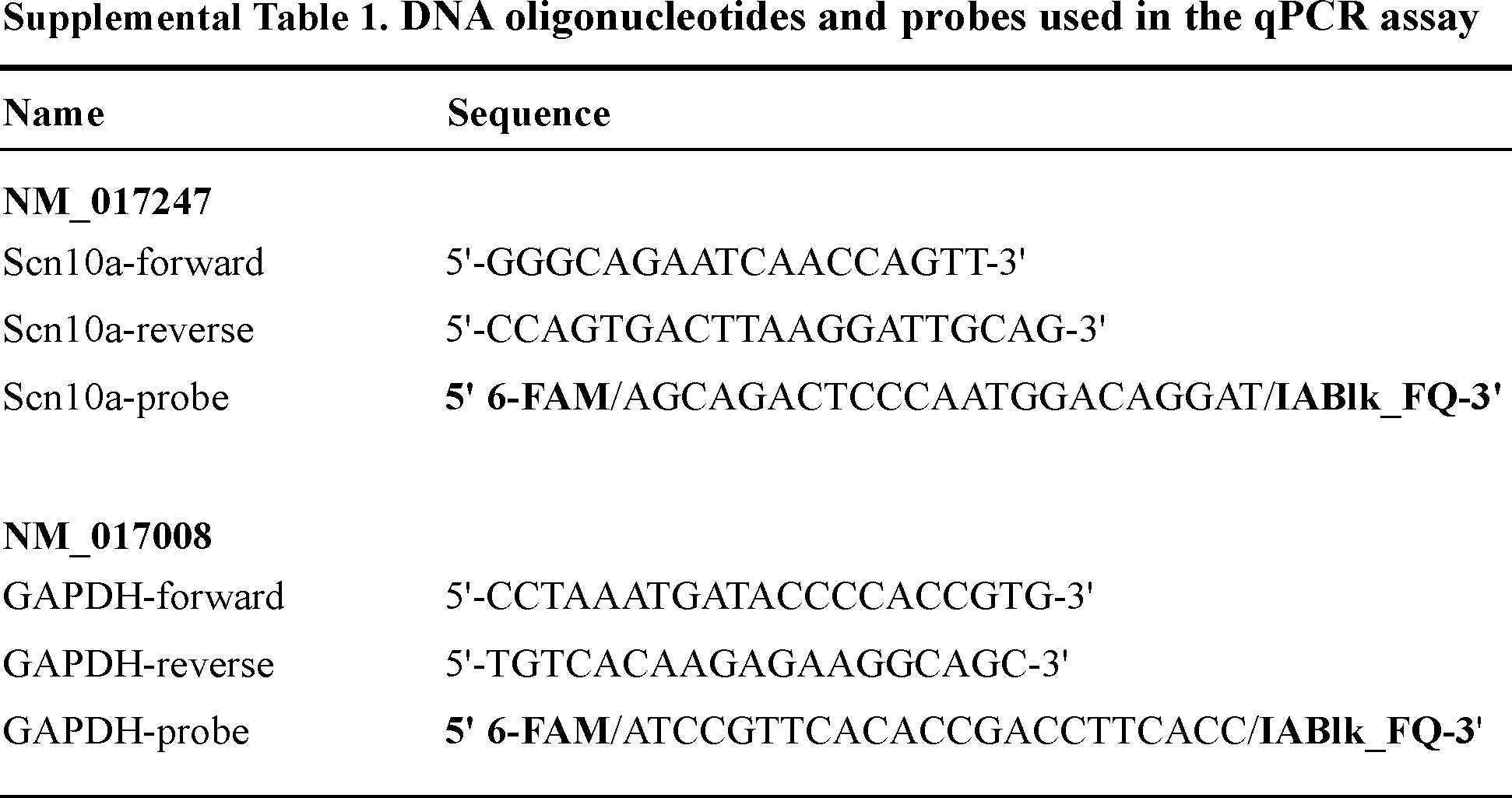

Supplement: Additional file 1: Table S1 — DNA oligonucleotides and probes used in the qPCR assay. [file 1742-2094-11-45-S1.tiff]
