# Supplementary material for: Functional up-regulation of Nav1.8 sodium channel in Aβ afferent fibers subjected to chronic peripheral inflammation
Source: J Neuroinflammation. 2014 Mar 7;11:45. doi: 10.1186/1742-2094-11-45 (PMC4007624; doi:10.1186/1742-2094-11-45)
Supplement: Additional file 2: Table S2 — Properties of INa currents in sham and inflamed large sensory neurons. V1/2act is the membrane potential for half-maximal channel activation. kact represents the slope factor for activation. *P <0.05, **P <0.01, and ***P <0.001 indicate statistically significant differences with sham group. $P <0.05 compared to large-sized DRG neurons extracted from rats 14 days post-CFA to day 8. Numbers in parentheses reflect numbers of recorded neurons. [file 1742-2094-11-45-S2.pdf]

	Peak I_{Na} currents (pA/pF)	$V_{1/2act}$ (mV)	k_{act}
**Sham**	90.72 ± 4.58 **(11)**	-32.66 ± 0.81	3.04 ± 0.41
**3d (n)**	122.09 ± 4,93*** **(11)**	-33,99 ± 1,92	2.33 ± 0.5
**8d (n)**	112,78 ± 5.24* **(8)**	-39.68 ± 1,63**	2.68 ± 0.41
**14d (n)**	135.43 ± 4.06***, $ **(8)**	-31.55 ± 0.82$	0.95 ± 0.08

Supplemental Table 2
