# Supplementary material for: Functional up-regulation of Nav1.8 sodium channel in Aβ afferent fibers subjected to chronic peripheral inflammation
Source: J Neuroinflammation. 2014 Mar 7;11:45. doi: 10.1186/1742-2094-11-45 (PMC4007624; doi:10.1186/1742-2094-11-45)
Supplement: Additional file 3: Table S3 — Mean peak sodium current amplitude, activation, and steady-state inactivation characteristics of Nav1.8 currents in sham and inflamed large sensory neurons. V1/2act and V1/2inact are the membrane potentials for half-maximal channel activation or inactivation, respectively. kact and kinact represent the slope factors for activation and inactivation. *P <0.05, **P <0.01, and ***P <0.001 indicate statistically significant differences with sham group. $$$P <0.001 and ###P <0.001 compared to large-sized DRG neurons extracted from rats 14 days post-CFA to days 3 and 8, respectively. Numbers in parentheses reflect numbers of recorded neurons. Ambroxol also significantly inhibits the leftward shift of the activation curves of Nav1.8 current (###P <0.001 compared to CFA-treated neurons). [file 1742-2094-11-45-S3.pdf]

	Peak $Na_v1.8$ currents (pA/pF)	$V_{1/2act}$ (mV)	k_{act}	$V_{1/2inact}$ (mV)	k_{inact}
**Sham (n)**	49.62 ± 2.4 **(13)**	-8.79 ± 1.01 **(13)**	2.68 ± 0.35	-33.6 ± 0.29 **(9)**	5.7 ± 0.09
**Day 3 (n)**	63.68 ± 6.04* **(6)**	-9.3 ± 0.23 **(6)**	1.77 ± 0.35	-39.9±1.34** **(7)**	6.49 ± 0.8
**Day 8 (n)**	72.56 ± 2.91*** **(7)**	-12.26 ± 1.61 **(7)**	3.2 ± 0.4	-38.4 ± 1.1* **(11)**	6.05 ± 0.26
**Day 14 (n)**	108.06 ± 1.52*** $$$, ### **(7)**	-20.25 ± 0.63*** $$$, ### **(7)**	2.39 ± 0.65	-56.3 ± 1.3*** $$$, ### **(9)**	6.49 ± 0.5
**Day 14 + ambroxol (20 µM) (n)**	10.68 ± 3.67### **(6)**	-8.52± 3.39### **(6)**	4.4 ± 0.61	NT	NT

Supplemental Table 3
